# Discovery of Novel Splice Variants and Regulatory Mechanisms for Microsomal Triglyceride Transfer Protein in Human Tissues

**DOI:** 10.1038/srep27308

**Published:** 2016-06-03

**Authors:** Takashi Suzuki, Larry L. Swift

**Affiliations:** 1 Department of Pathology, Microbiology and Immunology Vanderbilt University School of Medicine, Nashville, TN 37232, USA; 2 Research Service, Veterans Affairs, Tennessee Valley Health Care System, Nashville, TN, USA.

## Abstract

Microsomal triglyceride transfer protein (MTP) is a unique lipid transfer protein essential for the assembly of triglyceride-rich lipoproteins by the liver and intestine. Previous studies in mice identified a splice variant of MTP with an alternate first exon. Splice variants of human MTP have not been reported. Using PCR approaches we have identified two splice variants in human tissues, which we have named MTP-B and MTP-C. MTP-B has a unique first exon (Ex1B) located 10.5 kb upstream of the first exon (Ex1A) for canonical MTP (MTP-A); MTP-C contains both first exons for MTP-A and MTP-B. MTP-B was found in a number of tissues, whereas MTP-C was prominent in brain and testis. MTP-B does not encode a protein; MTP-C encodes the same protein encoded by MTP-A, although MTP-C translation is strongly inhibited by regulatory elements within its 5′-UTR. Using luciferase assays, we demonstrate that the promoter region upstream of exon 1B is quite adequate to drive expression of MTP. We conclude that alternate splicing plays a key role in regulating cellular MTP levels by introducing distinct promoter regions and unique 5′-UTRs, which contain elements that alter translation efficiency, enabling the cell to optimize MTP activity.

Microsomal triglyceride transfer protein (MTP) is an intracellular lipid transfer complex consisting of a unique 97 kD subunit, which possess lipid transfer activity, and the multifunctional 58-kD protein disulfide isomerase (PDI)^1^. *In vitro*, the complex facilitates the transfer of triglyceride, phospholipid, and cholesteryl ester between membrane vesicles^2^. Within the cell MTP is localized primarily within the endoplasmic reticulum (ER) of the cell^3^, presumably held within this compartment by a KDEL sequence on PDI^4^. A number of studies have shown that the KDEL sequence notwithstanding, MTP is also found in other locations within the cell^5^,^6^. Our laboratory has provided evidence that MTP is present within the Golgi complex in hepatocytes^7^ and adipocytes^8^ and localizes with lipid droplets within the cytoplasm of these cells^9^.

MTP is expressed in a number of tissues, including liver and intestine^10^,^11^,^12^,^13^,^14^, yolk sac^15^,^16^, testis and ovary^10^, retina^17^, adipose tissue^18^, placenta^19^, and kidney^10^,^20^. Whereas we know that MTP has the ability to transfer lipid within the cell, we do not know its specific function within different cells. In cells that express apolipoprotein (apo) B, such as liver, intestinal mucosa, cardiomyocytes, and yolk sac, MTP transfers lipid to apoB as it is translated and translocated into the lumen of the ER, initiating the assembly of a nascent lipoprotein particle^21^. In antigen presenting cells (APCs) as well as in adipocytes, MTP has been shown to lipidate the CD1 family of lipid antigen presenting proteins^22^,^23^. Recent studies in our laboratory have suggested that MTP is involved in lipid droplet maturation in adipocytes^9^.

Previous studies in our laboratory have shown that MTP exists as two isoforms (MTP-A, MTP-B) in mouse tissues^8^. MTP-A is the canonical form; MTP-B is a splice variant with a unique first exon (1B) located ~2.7 kb upstream of the first exon (1A) of MTP-A. The mature proteins, which differ only in the first 2–3 amino acids of the N-terminus, are equally effective in the assembly of apoB-containing lipoproteins; however, the tissue distribution is different with MTP-A being the predominant isoform in liver and intestine^8^, whereas MTP-B appears to be the major isoform in adipocytes as well as in professional APCs^8^,^24^.

At the time we discovered the splice variant of MTP in mouse tissues, we searched for an analogous splice variant in human tissues. We were unable to identify any sequence in the region 2–5 kb upstream of exon 1 on human chromosome 4 that was similar to the alternate first exon in the mouse genome. We were led to conclude that if there was an alternate human isoform, it was unlike that found in the mouse. Dougan *et al*. drew the same conclusion but went so far as to report that human MTP was not regulated via alternate splicing as is mouse MTP^24^. In this manuscript we report the identification of two splice variants in human *MTTP*. The first variant, which we have named MTP-B, has a unique first exon (1B) located 10.5 kb upstream of the first exon (1A) for canonical MTP, which we have re-named MTP-A. This transcript, while expressed in several tissues, does not express protein. The second variant, which we have named MTP-C, contains both exons 1B and 1A, is expressed in a number of tissues, and encodes MTP-A (or canonical MTP). We also demonstrate that elements within the extended 5′-UTR of MTP-C markedly suppress translation. Further investigation into the promoter regions of MTP-A and MTP-C revealed that the promoter for MTP-C can robustly drive the expression of MTP. We propose that MTP can arise through either splice variant and that the relative contribution from each is regulated by transcriptional and post translational elements.

## Results

### Identification of human MTP Splice Variants

**Figure 1:**
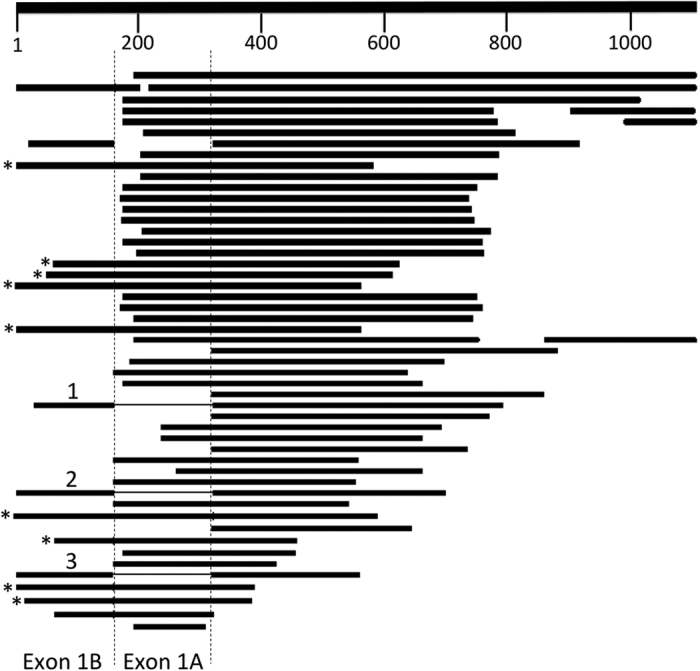
Alignment of 5′ end of human *MTTP* with sequences from EST database. The first 1100 bases in human *MTTP* (NM_000253) were blasted against the EST database. Sixty-four hits were observed. Fifteen ESTs aligned with a significant portion of the first exon (bases 1–160). Three ESTs (1–3) contained breaks in alignment with the *MTTP* mRNA that correspond perfectly with exon 1A. Nine ESTs (*) displayed significant alignment with the first three exons (1B, 1A, and 2).

**Figure 2:**
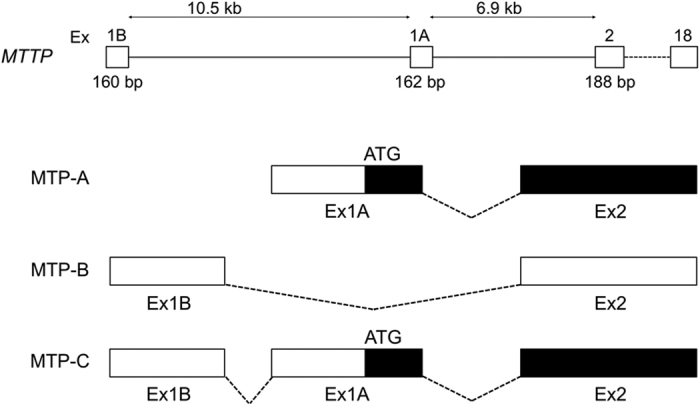
Diagram of human *MTTP* gene and slice variants. The human *MTTP* gene contains 19 exons and spans 40 kb. Exon 1B is located 10.5 kb upstream of exon 1A, which contains the initiator codon for canonical MTP (MTP-A). MTP-B arises through mRNA splicing of exon 1B to exon 2; MTP-C arises when exon 1A is not spliced out of MTP-B transcript. Solid (black) areas represent coding sequences. The MTP-B construct is apparently not translated as no protein is detected in MTP-B transfected cells.

Examination of NCBI reference sequences NM_000253.1, NM_000253.2, and NM_000253.3 revealed the latter two sequences contained an additional 160 nucleotides at the 5′-end of the message. Inspection of the human genomic sequence revealed that these 160 nucleotides matched a region of human chromosome 4, 10.5 kb upstream of the first coding exon for MTP. To explore the expression of this 5′ region in different tissues, we blasted the first 1100 bases of NM_000253.3 against the Expressed Sequence Tag (EST) database (NCBI;blastn). Multiple hits (64) were observed across the sequence (Fig. 1). We identified 15 ESTs that aligned with a significant portion of the first 160 bases in the *MTTP* mRNA sequence. Three of these sequences (1–3), derived from brain and testis, contained breaks in the alignment that corresponded perfectly with exon 2 (bp 161–322). In each of these three sequences, the first 160 nucleotides were spliced directly to exon 3, suggesting that this far upstream region represented an alternate first exon. Nine of the remaining sequences from testis, corpus callosum, and cerebellum displayed significant alignment with the first three exons of NM_000253.3. The results suggested the existence of two previously unidentified splice variants for human MTP. In keeping with the nomenclature we developed for mouse MTP, we will refer to exon 1 of the record (NM_000253.3) as 1B, exon 2 as exon 1A and exon 3 as exon 2, *etc*. (Fig. 2). In addition, we will refer to canonical human MTP as MTP-A (Exon 1A–2–18). The splice variant in which 1B is spliced directly to exon 2 will be designated MTP-B, and the transcript containing both exons 1B and 1A will be designated MTP-C.

### Tissue Expression of Splice Variants

**Figure 3:**
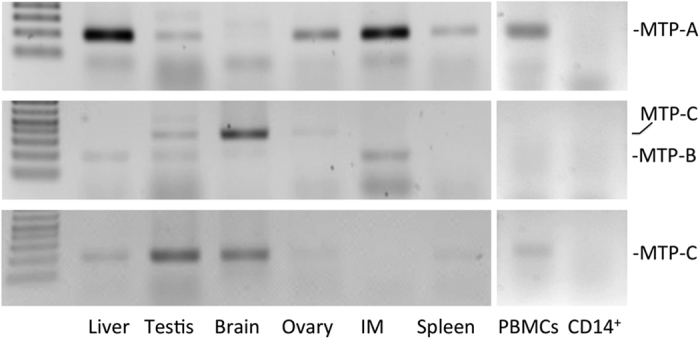
Expression of MTP splice variants in human tissues. MTP-A mRNA is robustly expressed in liver and intestinal mucosa (IM), but it is also found in testis, ovary, and spleen as well as in PBMCs. It is important to note that the product from the MTP-A primer set derives from both MTP-A and MTP-C transcripts. MTP-B mRNA was detected in liver, testis and intestinal mucosa. MTP-C specific primers revealed that MTP-C mRNA is expressed in liver, testis, brain, ovary, spleen, and PBMCs. (Note: primers for MTP-B also detect MTP-C.)

To confirm the presence of the splice variants in human tissues, we developed PCR primers for MTP-A, -B, and -C (Supplementary Table SI). As expected MTP-A was robustly expressed in liver and intestinal mucosa (IM); it was also found in testis, brain, ovary and spleen, as well as in peripheral blood mononuclear cells (PBMCs), but not in CD14^+^ monocytes (Fig. 3, top). Whereas we expect MTP-A mRNA to be most prominent in these tissues, we cannot eliminate contribution from MTP-C mRNA as the primers will amplify product from both transcripts. MTP-B was found in liver, testis, brain, and IM (Fig. 3, middle). MTP-C was prominent in brain and testis, but it was also present in liver, ovary and spleen and PBMCs (Fig. 3, bottom). Although MTP has been reported to be present in APCs^22^, neither MTP-A nor MTP-C was detected in CD14^+^ monocytes. Interestingly, Dougan *et al*. report MTP in human “monocyte derived dendritic cells”^24^ as well as in human monocyte cell lines^22^, but not in monocytes, suggesting that MTP may be expressed in more mature cells derived from monocytes, but not in primary monocytes themselves. The MTP-B and MTP-C PCR products generated from liver and testis were sequenced using both forward and reverse primers. The results clearly demonstrated that exon 1B was spliced directly to exon 2 in MTP-B, and MTP-C contained both exons 1B and 1A. In addition, the sequence of the fragments generated using the MTP-B and -C primers from testis and liver were compared and were completely identical.

### Expression of human MTP-B and -C

**Figure 4:**
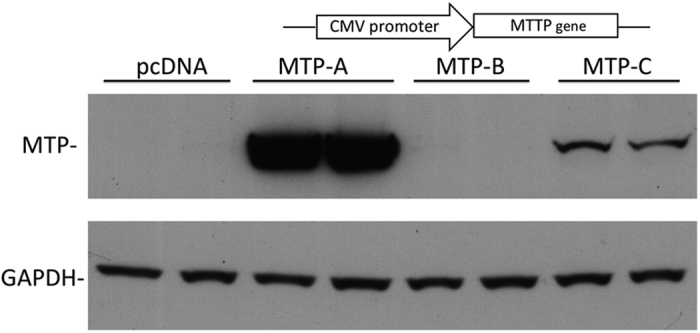
Cellular expression of human MTP splice variants. MTP-A, -B, and -C were cloned into the pcDNA3.1 vector (see schematic at top) and transfected into HEK 293 cells. Three days post transfection, equal amounts of cell lysate were separated by SDS-PAGE. MTP protein was detected by immunoblot and quantitated by densitometry using GAPDH as load control. No protein was detected from MTP-B. MTP protein levels in MTP-C transfected cells were ≈10% of the MTP protein levels in MTP-A transfected cells.

Human MTP-A, MTP-B, and MTP-C transcripts were cloned into pcDNA3.1 vectors, and equivalent amounts of DNA were transfected into HEK 293 cells. Three days post transfection, cell lysates were probed for MTP by immunoblotting (Fig. 4). Cells transfected with MTP-A expressed robust levels of the protein; however, we were unable to demonstrate any significant protein expression in cells transfected with MTP-B in HEK 293 cells or any other cell line. This was somewhat surprising given the fact that there are two potential translation initiation sites in exon 1B that are in frame with the main coding sequence for MTP. Cells transfected with the MTP-C construct expressed protein, but the levels were only approximately 10–12% of those seen in cells transfected with MTP-A. To eliminate the possibility that the decrease in protein expression observed in cells transfected with MTP-C was related to transfection efficiency of the transcript and/or mRNA production, we measured MTP mRNA levels in CHO cells transfected with each of the transcripts after DNase treatment (Supplement Fig. S1). As can be seen there was no difference in MTP mRNA levels between the cells transfected with the different transcripts.

### Triglyceride Transfer Activity

Triglyceride transfer activity was assessed in lysates of HEK 293 cells transfected with MTP-A, MTP-B or MTP-C using a fluorescence-based assay (Supplementary Fig. S2). Transfer activity was detected in cells transfected with either MTP-A or MTP-C; the activity observed with MTP-B transfected cells was not significantly different from cells transfected with an empty vector, consistent with the observation that little MTP protein, if any, is expressed in these cells (Fig. 4). The results clearly show that the protein produced by MTP-C construct is functional in lipid transfer. The level of transfer activity found in the MTP-C transfected cells relative to that found in MTP-A transfected cells was greater than might have been predicted, given the relative levels of MTP protein observed (Fig. 4). Given the robust expression of MTP-A, we speculate that the assay is rapidly saturated; hence, the relative levels of activity would not parallel the relative levels of protein.

### Analysis of 5′-untranslated region

**Figure 5:**
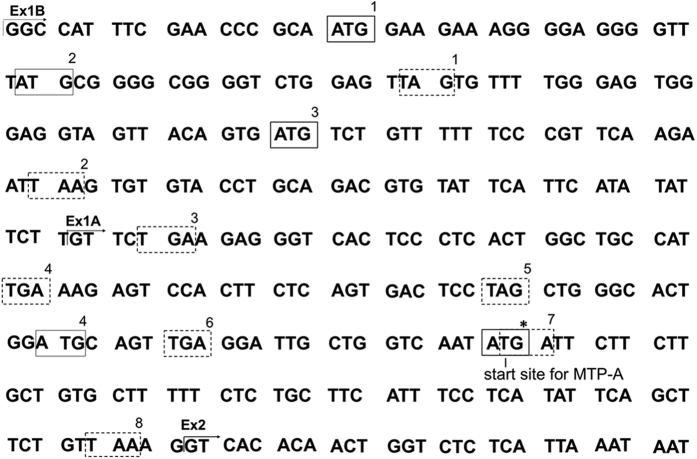
Nucleotide sequence for exons 1B and 1A. Start (boxes with solid lines) and stop (boxes with dashed lines) codons in the 5′-UTR of MTP-C are highlighted. Start sites ATG-1 and -3 are in frame with TGA-4; ATG-2 is in frame with TAG-1; ATG-4 is in frame with a stop codon at the end of exon 1A (TAA-8). ATG* denotes translation initiation site for MTP-A.

To explore the differences in MTP protein levels in cells transfected with MTP-A or MTP-C, we focused on the 5′-UTR of the MTP-C transcript (Fig. 5). Examination of this region revealed four translation initiation sites (ATGs) before the start site for MTP-A (ATG*). Three of the ATGs are in exon 1B. Two of these start sites (ATG-1, -3) are in frame with the main coding sequence for MTP; however, they are also in frame with three stop codons in exon 1A (TGA-4, TAG-5, and TGA-6), any of which would halt translation. The other start site in exon 1B (ATG-2) is out of frame with the main coding sequence, but is in frame with a stop codon 18 nucleotides downstream (TAG-1). The only upstream start site in exon 1A (ATG-4) is also out of frame with the main coding sequence but it is in frame with a stop codon in the main coding sequence (TAA-8). Thus, within the 5′-UTR, there are four potential upstream open reading frames. The only viable start site for the translation of MTP from the MTP-C construct is the same start site as for MTP-A (ATG*) (See also Fig. 2).

**Figure 6:**
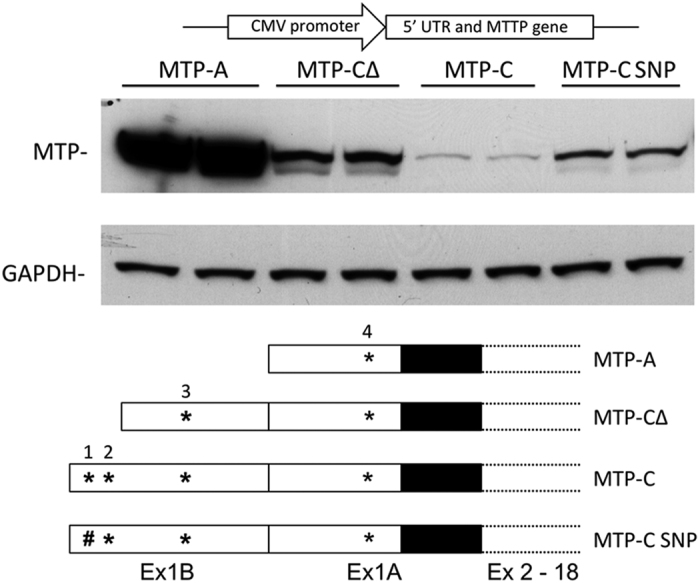
Effect of upstream ATGs on protein expression. HEK293 cells were transfected with human MTP-A, MTP-C, MTP-CΔ (MTP-C minus ATG-1 and -2, Fig. 5), MTP-C (SNP) (ATG-1, Fig. 5, mutated to ATA), and cellular protein harvested 72 hours later. A schematic of the vectors used is shown at the top. MTP protein was detected by immunoblot and quantitated by densitometry using GAPDH as load control. *uATG; ^#^SNP rs11944752, ATG > ATA.

To explore further the possibility that upstream ATGs (uORFs) regulate the expression of MTP, we transfected HEK 293 cells with human MTP-A, MTP-C, MTP-CΔ (MTP-C minus ATG-1 and ATG-2 in exon 1B, Fig. 5), and MTP-C (SNP) (ATG-1 (Fig. 5) in exon 1B changed to ATA). This change corresponds to a SNP in human MTP (rs11944752) that has been associated with plasma glucose and insulin (MAGIC Consortium HGVM 4589669) and cholesterol levels^25^. Cells transfected with MTP-A expressed robust levels of protein (Fig. 6). MTP expression was markedly reduced in cells transfected with MTP-C, similar to that seen in Fig. 4. Removal of the first two uATGs (MTP-CΔ) resulted in increased levels of protein compared with MTP-C. Interestingly, MTP expression in cells transfected with MTP-C(SNP) were similar to those found with MTP-CΔ. The results suggest that MTP expression is regulated by the presence of the uATGs.

### Analysis of Promoter Regions

**Figure 7:**
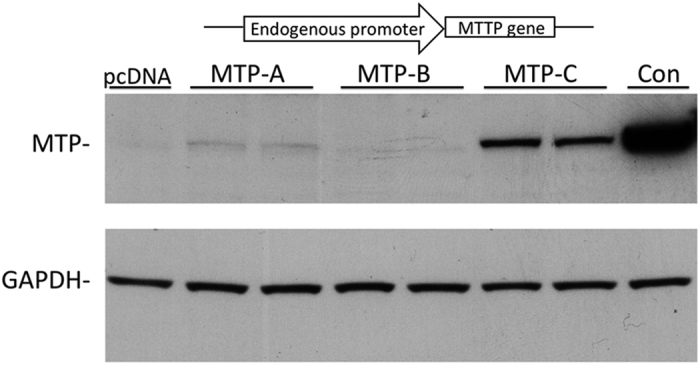
Effect of promoter regions on MTP protein expression. The 2 kb region upstream of the alternate exon 1B was cloned into MTP-B and MTP-C expression vectors (pcDNA) in which the CMV promoter had been removed. In addition, the 2 kb region upstream of exon 1A was cloned into the MTP-A expression vector (pcDNA minus the CMV promoter region). The vectors were transfected into Caco2 cells. A schematic of the vectors used is shown at the top. Three days post transfection, MTP protein levels were assessed by immunoblotting.

The discovery of the alternate transcript with a different first exon, led us to investigate the promoter regions of these transcripts. We cloned 2 kb regions upstream of the alternate exon (1B) into the MTP-B and MTP-C expression vectors (pcDNA) in which the CMV promoter had been removed. In addition, we cloned the 2 kb region upstream of exon 1A into the MTP-A expression vector (pcDNA minus the CMV promoter). The three vectors were transfected into CHO cells, and MTP expression monitored by immunoblotting 3 days post transfection. Similar to the results with the CMV-promoter driven constructs (Fig. 4), we saw no protein expression with the MTP-B construct. Surprisingly, we found robust expression of protein with the MTP-C construct, but no detectable protein with the MTP-A construct. This led us to question if CHO cells might lack a specific transcription factor necessary to transcribe the MTP-A construct. We then transfected the constructs into Caco2 cells and found relatively similar results as with CHO cells (Fig. 7). MTP expression was markedly increased in cells transfected with the MTP-C construct. In cells transfected with the MTP-A construct, we saw a detectable, but very small increase in protein over background levels. Consistent with previous results, the MTP-B construct did not produce any protein.

**Figure 8:**
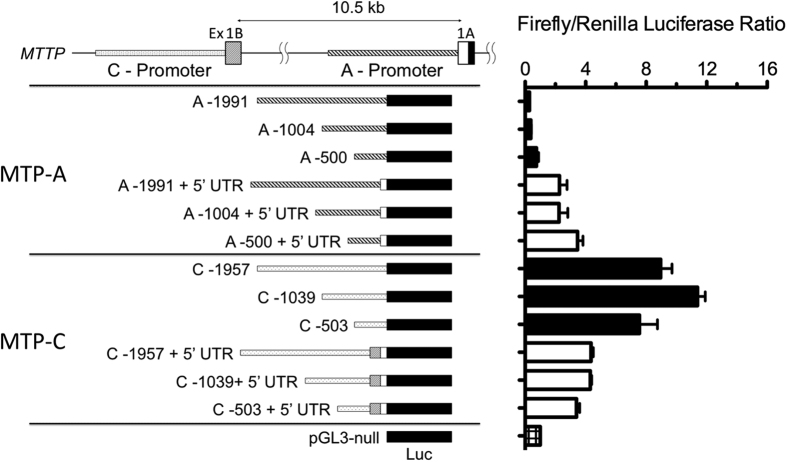
Analysis of promoter regions. Promoter regions (2 kb, 1 kb, 0.5 kb) for MTP-A (A-1991, A-1004, A-500) and MTP-C (C-1957, C-1039, C-503) were cloned into the pGL3-Control vector from which the SV40 promoter had been removed. Additional pGL3-Control vectors that contained the 5′-UTR for MTP-A and -C (+5′-UTR) as well as the respective promoter regions were constructed. The vectors were transfected into HEK 293 cells and assayed for luciferase activity. Data are expressed as mean ± s.d. (n = 3/group).

The results suggested differences in the two promoter regions. To explore this in more detail we cloned promoter regions (2 kb, 1 kb, and 0.5 kb) for MTP-A and MTP-C into the pGL3-Control vector from which the SV40 promoter had been removed. Given our results suggesting that the 5′-UTR might also affect translation, we developed vectors which contained, in addition to the promoter region, the respective 5′-UTR regions. These constructs were then transfected into HEK 293 cells and assayed for luciferase activity as described below (Fig. 8). The promoter regions for MTP-C led to an 8–10 fold increase in luciferase activity compared with that seen with the respective promoter regions for MTP-A without or with the 5′-UTR (p < 0.001). There were no apparent differences in activity with the different promoter lengths in any of the constructs, suggesting that the 0.5 kb regions for both MTP-A and -C contained the main promoter regulatory elements. Inclusion of the 5′-UTR in the MTP-A construct significantly increased luciferase activity only for the 0.5 kb promoter (p < 0.05); in contrast, inclusion of 5′-UTR in the MTP-C construct decreased luciferase activity for each of the promoter regions (p < 0.001).

## Discussion

Previous studies in our laboratory identified a splice variant of mouse MTP, which contained a unique first exon (1B) located approximately 2.7 kb upstream of the first exon (1A) for canonical MTP (MTP-A)^8^. At the same time Dougan *et al*.^24^ reported the discovery of the alternate isoform of mouse MTP. We reported that expression of the MTP isoforms was tissue-specific with MTP-A being the prominent isoform in apoB-expressing tissues such as liver and intestine, whereas MTP-B was more prominent in adipose tissue. Dougan and coworkers made a similar observation that the alternate isoform, which they called MTPv1, was expressed in non-apoB secreting tissues, including thymocytes and antigen presenting cells (APCs)^24^. They also demonstrated that MTPv1 is the major transcript in hematopoietic cells. The difference in tissue distribution of the MTP isoforms suggests that alternative splicing plays a role in the regulation of MTP expression, and this is achieved by alternate promoters driven by tissue-specific transcription factors. A major unresolved question related to the presence of alternate isoforms in human tissue and the possibility that MTP expression in human tissues was regulated in a manner similar to its regulation in mouse tissue. The studies in this paper report the discovery of MTP splice variants in human tissues and demonstrate that human MTP is also regulated by alternative splicing.

The human *MTTP* gene was first characterized by Sharp *et al*.^26^ and shown to consist of 18 exons and span 55–60 kb. Consistent with this report, the earliest NCBI reference sequences (NM_000253.1) reported 18 exons with coding beginning in the first exon. Subsequent entries (NM_000253.2, NM_000253.3), using data derived from the expressed sequence tag (EST) database, included an additional 160 nucleotides at the 5′ end of the sequence (NM_000253.2, NM_000253.3), which matched a region of human chromosome 4, 10.5 kb upstream of the first coding exon (1A) for MTP. We concluded that these 160 nucleotides represented an additional exon for human *MTTP*. By blasting the 5′-region of *MTTP* against the human expressed sequence tag database, we identified ESTs in which this additional exon (1B) was spliced directly to exon 2, as well as ESTs that contained exons 1B, 1A and 2. We named the former splice variant MTP-B and the latter MTP-C. RT-PCR confirmed the presence of these constructs in human tissues/cells (Fig. 3).

We were unable to document protein production from the MTP-B construct in CHO, HEK 293 or Caco2 cells, which is a bit surprising given that there are two potential translation initiation sites within exon 1B, both of which are in frame with the main coding sequence (CDS). In addition, one of these sites is situated in the context of a strong Kozak consensus sequence. Nevertheless, we have been unable to detect any protein from cells transfected with this construct. It is possible that exon 1B is important as part of the 5′-UTR in MTP-C, and the splicing of exon 1B to exon 2 (formation of MTP-B) occurs only in the context of formation of MTP-C within the cell. The MTP-C transcript clearly expresses protein (Figs 4 and 7). The additional exon (1B) at the 5′ end of the mRNA introduces three potential translation initiation sites; however, the only viable start site in this construct is the start site for canonical MTP (or MTP-A). Thus, in human tissues MTP can be produced from either of two constructs that arise via alternative splicing.

Our data indicate that MTP expression from the MTP-C transcript is regulated, in part, by elements within the extended 5′-UTR of the transcript, specifically by upstream translation initiation sites (ATGs) that may, in some cases, be associated with upstream open reading frames (uORFs). uORFs are sequences with an initiator codon in frame with a termination codon either upstream or downstream of the initiation site for the primary ORF. uORFs are generally associated with decreased protein expression levels, because they reduce the efficiency of translation of the downstream ORF. Protein levels may be reduced from 30–80%^27^ depending on a number of factors including the strength of the Kozak sequence^28^ surrounding the translation initiation site. In the case of weaker Kozak sequences the ribosome might read the ATG or move to a start site further downstream. If the ATG is recognized, it may translate the uORF and dissociate, translate the uORF and stall creating a blockade that may trigger mRNA decay, or translate the uORF and then re-initiate to translate the primary ORF. In any case, translation efficiency is reduced, and protein expression is decreased. The presence of the uATGs in the MTP-C construct clearly have an effect on translation (Fig. 8) and protein expression (Fig. 6). Removal of the first two ATGs in exon 1B (MTP-CΔ) leads to increased protein production compared to MTP-C (Fig. 6). In addition, mutation of the first ATG (ATG-1 in Fig. 5) to ATA, corresponding to a SNP (rs11944752), also increases protein expression. Finally, the luciferase experiments (Fig. 8) suggest that the presence of the 5′-UTR significantly reduces translation efficiency. Thus, we conclude that MTP expression from the MTP-C transcript is regulated, in part, by elements in the 5′-UTR. Interestingly, the SNP rs11944752 has been associated with fasting glucose and insulin levels (MAGIC Consortium HGVM 4589669) and total and LDL cholesterol^25^. It is not difficult to see how alterations in the expression of MTP could affect total and LDL cholesterol levels. MTP is essential for the assembly and secretion of very low density lipoprotein (VLDL) by the liver^21^, and VLDL is the sole precursor of low density lipoproteins (LDL), which transport ~70% of plasma cholesterol in humans. Tietge *et al*. have shown that increasing hepatic MTP levels leads to dramatic increases in hepatic VLDL secretion and plasma triglyceride levels in mice^29^. Our studies have shown that the SNP, which abolishes an uATG, leads to increased expression of MTP. *In vivo*, this could lead to increased VLDL assembly and secretion and increased plasma cholesterol levels. A connection between the SNP and fasting glucose and insulin levels is a little more difficult, as to date we are unaware of metabolic evidence connecting MTP with glucose metabolism. Importantly, the SNP association clearly suggests that MTP encoded from the MTP-C transcript is of biologic and physiologic importance, especially in the maintenance of plasma lipoprotein levels.

In addition to differential translational regulation of the two transcripts, our studies also demonstrate differences in transcriptional regulation (Figs 7 and 8). Promoters for the MTP-A and -C transcripts are localized within the first 500 bp upstream of the transcription start site as we found no difference in luciferase activity with promoter regions larger than 0.5 kb (Fig. 8). Several studies have provided evidence that the major promoter elements for canonical human MTP (MTP-A) reside in the first 150 bp upstream of the transcription start site^30^. Within this region are positive (hepatic nuclear factor [HNF]-1, HNF-4, direct repeat [DR]1 and FOX) and negative (SRE/IRE) regulatory elements. *In silico* examination of the 500 bp upstream of the transcription start site for MTP-A and -C using PROMO software^31^,^32^, revealed several common lipogenesis/adipogenesis-related transcription factor binding sites, including C/EBPα^33^, C/EBPβ^33^,^34^ and XBP-1^34^,^35^. An ELK-1 binding site, located approximately 300 bp upstream of the transcription start site, is unique to the MTP-C promoter region. Three ELK-1 ChIP-seqs in the human cell lines K562, HeLa, and gm12878 have been published as a part of series GSE31477 in the NCBI Gene Expression Omnibus. All three cell lines exhibit binding of ELK1 within the promoter for MTP-C^36^. Wang *et al*. reported that the Med23 subunit of the Mediator Complex and its binding partner ELK-1 are critical regulators of adipogenesis^37^. Further investigation and analysis of the MTP-C promoter region may provide support for a biological role of MTP-C in lipogenesis/adipogenesis.

It is interesting to compare the structures of mouse and human *MTTP* gene. Whereas the two genes are highly homologous from exons 1A through 18 with 16 of the 18 exons being identical in size, the structures of the two genes upstream of exon 1A are different. Mouse *Mttp* has an alternate first exon (1B) ~2.7 kb upstream of exon 1A^8^,^24^; however, we have been unable to identify a counterpart to this exon in human *MTTP*. According to public databases (e.g., UCSC Genome Browser) mouse *Mttp* contains two additional exons ~4.8 and 9.0 kb upstream of exon 1B. We have confirmed the presence of these exons by 5′-RACE techniques. We have also confirmed the presence of two exons upstream of exon 1A in human *MTTP*. Exon 1B, reported in this paper, is ~10.6 kb upstream of exon 1A. A second exon is ~3.9 kb upstream of exon 1A; however, this exon, if it is spliced to exon 2, does not contain a start codon in-frame with the main coding sequence of MTP. It is possible that it splices to exon 1A, making another MTP-C-like transcript from which translation starts from the MTP-A start site, but we have no evidence that this occurs.

In conclusion, our studies have identified a unique splice variant of human MTP, which we have named MTP-C. This variant contains 19 exons, including a unique exon (1B) located ~10 kb upstream of the first exon (1A) for canonical MTP (MTP-A). This transcript encodes the same MTP protein as does the MTP-A transcript. The extended 5′-UTR of MTP-C mRNA includes several translation initiation sites that suppress translation of the main ORF, decreasing protein production. A SNP that abolishes one of the start sites and is associated with fasting glucose/insulin levels and total and LDL cholesterol, underlines the potential significance of the MTP-C transcript in carbohydrate and lipid metabolism. In addition, the two transcripts are differentially regulated by distinct promoter regions. It is our hypothesis that regulation of MTP expression from the MTP-C splice variant through transcriptional and translational mechanisms provides a way for the cell to maintain MTP levels within a critical concentration range, maximizing its tissue-specific function. Elucidation of the function of MTP in tissues that express MTP-C will be critical in testing this hypothesis.

## Methods

### Antibodies and reagents

Rabbit anti-MTP was developed in our laboratory and has been described previously^7^.

### Human tissue

De-identified human tissue was obtained through the Cooperative Human Tissue Network (CHTN), Vanderbilt University Medical Center. The Institutional Review Board determined this study did not qualify as “human subject” research per §46.102(f)(2). Consequently, informed consent was not required (IRB# 151681). Human peripheral blood mononuclear cells (PBMCs) were isolated from fresh whole blood (5 mM EDTA) by Ficoll density gradient centrifugation. CD14^+^ monocytes were positively selected from PBMCs using magnetically labeled CD14 MicroBeads (Miltenyi Biotec, Germany) as per manufacturer’s instructions. Obtaining human blood for these studies was approved by the Institutional Review Board at Vanderbilt University Medical Center (#070416), and written informed consent was obtained from all participants. All experiments were performed in accordance with relevant guidelines and regulations.

### Cell culture

CHO cells were purchased from American Type Culture Collection (Rockville, MD) and cultured in Kaighn’s modification of Ham’s F-12 (F12K) media containing 10% fetal bovine serum (FBS). HEK 293 cells were cultured in Dulbecco’s Modified Eagle’s Medium (Corning Life Sciences, Tewksbury, MA) supplemented with 10% FBS. Caco2 cells HepG2 cells were cultured in Eagle’s Minimum Essential Media supplemented with 10% FBS.

### Isolation of RNA and genomic DNA and cDNA synthesis

Total RNA of tissues and cells was isolated using Trizol reagent (Life Technologies, Grand Island, NY) according to the manufacture’s instruction. cDNAs were synthesized using Super Script III Reverse Transcriptase (Life Technologies) according to the manufacturer’s instructions. In brief, 1 μg of total RNA was combined with random hexamers (Life Technologies) and oligo d(T)_16_ (Life Technologies) (both 1.25 μM final concentration) and dNTP mix (Sigma-Aldrich, 0.5 mM final concentration). The mixture was heated at 65 °C for 5 min and then quickly chilled on ice for 5 min. 5X first strand reaction buffer, DTT (10 mM final concentration) and Super Script III enzyme (100 U) were then added, and the mixture was incubated at 25 °C for 5 min, 50 °C for 60 min, and 70 °C for 15 min. HEK 293 genomic DNA was purified with DNeasy Tissue Kit (QIAGEN, Venlo, Netherlands) according to the manufacture’s instruction. To remove vector DNA from total RNA extracts, the extracts were treated with DNase I (New England Biolabs, Ipswich, MA) as follows. Total RNA (1 μg) was incubated with 2 unit DNase I (50 μl total volume) for 15 min at 37 °C. DNase I treated RNA (5 μl) was used for the cDNA synthesis step.

### Primer sequences for semi-quantitative RT-PCR and cloning

Supplementary Table S1 shows the sequences of primer sets for semi-quantitative RT-PCR. PCR cycle conditions were: 95 °C for 30 s, 58 °C for 30 s, 72 °C for 30 s with 35 cycles. Semi-quantitative RT-PCR was performed with GoTaq DNA polymerase.

Supplementary Table S2 shows the primer sequences used for PCR cloning of MTP genes. Liver cDNA was used as template DNA for MTP-A, and brain cDNA was used for MTP-B and MTP-C. Easy-A High-Fidelity PCR Cloning enzyme (Agilent Technologies, Santa Clara, CA) was used to amplify full length MTP cDNAs. PCR cycle conditions were: 95 °C for 30 s, 68 °C for 10 min with 32 cycles. PCR products were ligated into pGEM-T easy vector (Promega, Madison, WI) and MTP sequences were confirmed. Using restriction enzyme sites in pGEM-T easy vector (Not I) or PCR primers (Xho I, Bam HI), full length MTP cDNAs were re-ligated into pcDNA3.1 (−) vector (Life Technologies). To make MTP-A full Ex1a vector, MTP-C expression vector with all uATGs, and MTP-C SNP vector, PCR was performed with Easy-A High-Fidelity PCR Cloning enzyme with the following conditions: 95 °C for 40 s, 60 °C for 30 s, 72 °C for 1 min with 30 cycles. PCR products were ligated into pGEM-T easy vector (Promega, Madison, WI). After MTP sequences were confirmed, vectors were digested with Eco RI (in pGEM-T easy vector) and Nhe I (MTP-A) or Pst I (MTP-C) restriction enzymes and subcloned into MTP-A or MTP-C expression vectors.

The primer sequences used for cloning MTP promoter regions are shown in Supplementary Table S3. HEK 293 genomic DNA was used as template DNA. PCR were performed with Easy-A High-Fidelity PCR Cloning enzyme with the following conditions: 95 °C for 30 s, 68 °C for 10 min with 32 cycles. PCR products were digested with Bgl II (in Forward Primer) and Bse YI (MTP-A) or Pst I (MTP-B and MTP-C) and re-ligated to CMV promoter region removed MTP expression vectors.

Supplementary Table S4 shows the primer sequences used for cloning of promoter ±5′ UTR of MTPs. The preimer sets contained a Kpn I site in the forward primer and a Hind III site in the reverse primer, used for subcloning into the pGL3-Control vector in which the SV40 promoter region had been removed. To make promoter-less pGL3 vector (pGL3-null), the pGL3-Control vector was digested with Bgl II and Hind III restriction enzymes and re-ligated with site change (Bgl II to Hind III) linker.

### Isolation of Cell Protein

Cells were solubilized in 20 mM HEPES (pH 7.4), 1.0 mM EGTA, 1% Triton X-100, and 10% glycerol on ice for 20 min. The extracts were then centrifuged at 4 °C for 5 min at 14,000 × g in an Eppendorf microfuge. The supernatant was recovered, and protein concentration was determined using the bicinchoninic acid (BCA) method (Thermo Fisher Scientific, Waltham, MA). Aliquots were taken for SDS-PAGE and immunoblotting as described below.

### SDS-polyacrylamide gel electrophoresis and immunoblotting

Samples were solubilized in NuPAGE LDS sample buffer and separated by SDS-PAGE using NuPAGE bis-tris gels (4–12% gradients) (Life Technologies) with morpholinepropanesulfonic acid SDS running buffer^38^. The proteins were transferred to nitrocellulose membranes. The membranes were blocked in TBS with 5% non-fat milk, incubated overnight at 4 °C with primary antibody, washed extensively, and incubated for 1 hr at room temperature with the appropriate secondary antibody conjugated with horseradish peroxidase. Bands were visualized using Western Lightning^®^ Plus-ECL enhanced chemiluminescence substrate (Perkin Elmer, Waltham, MA) and quantitated by densitometry (BioRad Model GS-700 Imaging Densitometer) equipped with Quantity One software.

### Triglyceride transfer activity

Triglyceride transfer activity was assessed using a fluorescent-based kit assay^39^ (Chylos, Inc., Woodbury, NY). CHO cells were transfected with mouse MTP-A, -B, or -C as described above. Three days post transfection the cells were lysed, and aliquots of the lysate were used in the transfer assay and for protein determination (BCA). The transfer assays were run for 5 h, and the results were expressed as the percent triglyceride transferred per microgram of cell protein. Lysate from HepG2 cells was used as a positive control. CHO cells transfected with an empty pcDNA vector served as a negative control.

### Translation luciferase assays

The following 5′-untranslated regions (UTRs) were subcloned downstream of an SV40 promoter and upstream of the firefly luciferase gene in a pGL3-Control vector (Promega): 1) 5′-UTR for MTP-A; 2) 5′-UTR for MTP-A plus first four bases (ATGA) of coding sequence of MTP; 3) 5′-UTR for MTP-C; 4) 5′-UTR for MTP-C plus first four bases (ATGA of coding sequence of MTP. HEK 293 cells were transfected with pGL3-Control or one of the 5′-UTR pGL3 reporter constructs (above), along with pRL-TK renilla luciferase construct (Promega) using FuGENE 6. The cells were harvested three days after transfection and assayed for luciferase activity using a Dual-Glo Luciferase Assay System (Promega).

## Additional Information

**How to cite this article**: Suzuki, T. and Swift, L. L. Discovery of Novel Splice Variants and Regulatory Mechanisms for Microsomal Triglyceride Transfer Protein in Human Tissues. *Sci. Rep.* **6**, 27308; doi: 10.1038/srep27308 (2016).

## Supplementary Material

Supplementary Information
