# Supplementary material for: Discovery of Novel Splice Variants and Regulatory Mechanisms for Microsomal Triglyceride Transfer Protein in Human Tissues
Source: Sci Rep. 2016 Jun 3;6:27308. doi: 10.1038/srep27308 (PMC4891672; doi:10.1038/srep27308)
Supplement: Supplementary Information [file srep27308-s1.pdf]

Supplementary Information

Discovery of novel splice variants and regulatory mechanisms for microsomal triglyceride transfer protein in human tissues

Takashi Suzuki[1*], Larry L. Swift[1,2]

[1]*Department of Pathology, Microbiology and Immunology*

Vanderbilt University School of Medicine, Nashville, TN 37232

[2]*Research Service, Veterans Affairs, Tennessee Valley Health Care System, Nashville, TN*

Supplementary Figure S1. MTP mRNA levels in CHO cells transfected with human MTP constructs.

Supplementary Figure S2. Triglyceride transfer activity

Supplementary Table S1. Sequences of primer sets for semi-quantitative RT-PCR of human MTTP mRNA

Supplementary Table S2. Sequences of primer sets for cloning of human MTTP genes

Supplementary Table S3. Sequences of primer sets for cloning of human MTTP gene promoter regions

Supplementary Table S4. Sequences of primer sets for cloning of uORFs and promoter ± uORF of MTTP genes

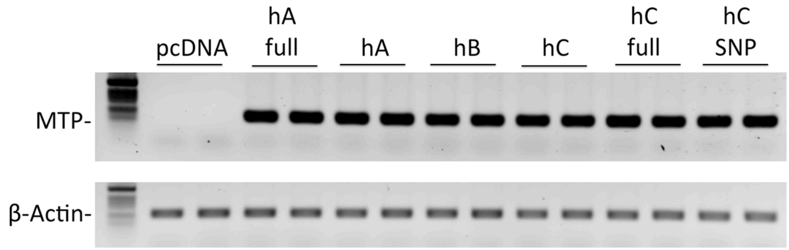

Supplementary Figure S1. **MTP mRNA levels in CHO cells transfected with human MTP constructs.** CHO cells were transfected with human MTP transcripts, and three days post transfection, total RNA was isolated. The RNA extract was treated with DNase I as described in Methods. Total MTP mRNA levels were assessed by RT-PCR using the primers shown in Table S1. hA, hB, hC represent the coding sequence of the three variants; hA full, hC full – represent the coding sequence plus the 5'-UTR for MTP-A and -C; hCSNP represents hC full in which ATG-1 (Fig. 5) was mutated to ATA.

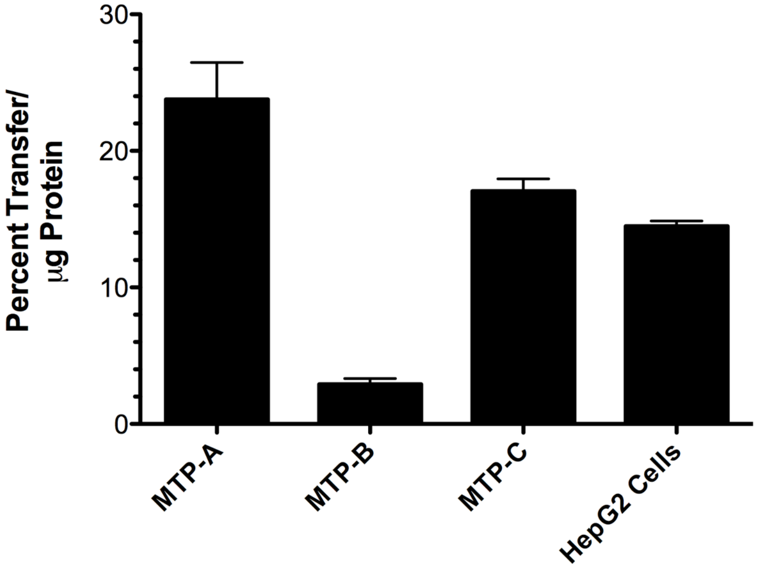

Supplementary Figure S2. **Triglyceride transfer activity.** CHO cells were transfected with human MTP-A, -B, and -C. Three days post transfection the cells were lysed and aliquots of the lysate taken for the fluorescence based triglyceride transfer assay (Chylos, Inc.). The assays

were run for 5 h, and the results are expressed as the percent of total triglyceride transferred/microgram cell protein. Data are expressed as mean ± s.d. (n = 3/group).

Supplementary Tables

Table S1. Sequences of primer sets for semi-quantitative RT-PCR of human MTTP mRNA

MTTP mRNA	Forward	Reverse	Size
MTP-A	CAGTTGAGGATTGCTGGTCA	CACGCTGTCTTGCAGTTTTC	187 bp
MTP-B	GGAGGTAGTTACAGTGATGTC	CACGCTGTCTTGCAGTTTTC	187 bp
MTP-C	TGTTTTGGGAGTGGGAGGTA	TGACCAGCAATCCTCAACTG	195 bp
Total MTP	GAAGGCTGGTCTTCACGGTA	CCGTTGAAAAAGGTGACAGG	162 bp

Table S2. Sequences of primer sets for cloning of human MTTP genes

MTTP GENE	FORWARD	REVERSE
MTP-A	CCGCTCGAGATGATTCTTCTTGCTGTGCTTTTTC TCTGC	CGCGGATCCTCAAAACCATCCGCTGGAAGTACTATCC GGC
MTP-B	CGGGGTCTGGAGTTAGTGTT	CGCGGATCCTCAAAACCATCCGCTGGAAGTACTATCC GGC
MTP-C	CGGGGTCTGGAGTTAGTGTT	CGCGGATCCTCAAAACCATCCGCTGGAAGTACTATCC GGC
MTP-A full Ex1A	GTTCTGAAGAGGGTCACTCC	GGGGTCGTAAATCCAGACCT
MTP-C w/all uATG	GAACCCGCAATGGAAGAAAG	AATGAATACACGTCTGCAGGTACA
MTP-C SNP	GAACCCGCAATAGAAGAAAG	AATGAATACACGTCTGCAGGTACA

Table S3. Sequences of primer sets for cloning of human MTTP gene promoter regions

MTTP promoter region	FORWARD	REVERSE
MTTP-A promoter	TTAAGATCTTCATCTGTAATGAAACAATCTCCA	TGACCAGCAATCCTCAACTG
MTTP-B and -C promoter	TTAAGATCTGCAGCACTGCACTCCAACTA	AATGAATACACGTCTGCAGGTACA

Table S4. Sequences of primer sets for cloning of uORFs and Promoter ± uORF of MTTP genes

MTTP promoter ± 5' UTR	FORWARD	REVERSE
2 kb MTTP-A promoter	TTAGGTACCTCATCTGTAATGAAACAATCTCCA	CCTAAGCTTCTGCAACAATCATGTTTATCTTTG
1 kb MTTP-A promoter	TTAGGTACCATAGCCCTTTGCTGGCACAT	CCTAAGCTTCTGCAACAATCATGTTTATCTTTG
0.5 kb MTTP-A promoter	TTAGGTACCACATTATTTTGAAGTGATTGGTGGTG	CCTAAGCTTCTGCAACAATCATGTTTATCTTTG
2 kb MTTP-A promoter + 5' UTR	TTAGGTACCTCATCTGTAATGAAACAATCTCCA	CCTAAGCTTATTGACCAGCAATCCTCAACTGC
1 kb MTTP-A promoter + 5' UTR	TTAGGTACCATAGCCCTTTGCTGGCACAT	CCTAAGCTTATTGACCAGCAATCCTCAACTGC
0.5 kb MTTP-A promoter + 5' UTR	TTAGGTACCACATTATTTTGAAGTGATTGGTGGTG	CCTAAGCTTATTGACCAGCAATCCTCAACTGC
2 kb MTTP-B&C promoter	TTAGGTACCGCAGCACTGCACTCCAACTA	CCTAAGCTTTGGCCGCAGTTCGATGACG
1 kb MTTP-B&C promoter	TTAGGTACCCTCCTGACCTCAGGTGATCC	CCTAAGCTTTGGCCGCAGTTCGATGACG
0.5 kb MTTP-B&C promoter	TTAGGTACCATGGCAACCCCAAAACACAC	CCTAAGCTTTGGCCGCAGTTCGATGACG
2 kb MTTP-B&C promoter + 5' UTR	TTAGGTACCGCAGCACTGCACTCCAACTA	CCTAAGCTTATTGACCAGCAATCCTCAACTGC
1 kb MTTP-B&C promoter + 5' UTR	TTAGGTACCCTCCTGACCTCAGGTGATCC	CCTAAGCTTATTGACCAGCAATCCTCAACTGC
0.5 kb MTTP-B&C promoter + 5' UTR	TTAGGTACCATGGCAACCCCAAAACACAC	CCTAAGCTTATTGACCAGCAATCCTCAACTGC
